# A Phase II Clinical Trial of Pembrolizumab Efficacy and Safety in Advanced Renal Medullary Carcinoma

**DOI:** 10.3390/cancers15153806

**Published:** 2023-07-27

**Authors:** Chijioke Nze, Pavlos Msaouel, Mohamed H. Derbala, Bettzy Stephen, Abdulrahman Abonofal, Funda Meric-Bernstam, Nizar M. Tannir, Aung Naing

**Affiliations:** 1 Department of Lymphoma and Myeloma, The University of Texas MD Anderson Cancer Center, Houston, TX 77030, USA; ccnze@mdanderson.org; 2 Department of Genitourinary Medical Oncology, The University of Texas MD Anderson Cancer Center, Houston, TX 77030, USA; pmsaouel@mdanderson.org; 3 Department of Translational Molecular Pathology, The University of Texas MD Anderson Cancer Center, Houston, TX 77030, USA; 4 David H. Koch Center for Applied Research of Genitourinary Cancers, The University of Texas MD Anderson Cancer Center, Houston, TX 77030, USA; 5 Department of Investigational Cancer Therapeutics, The University of Texas MD Anderson Cancer Center, Houston, TX 77030, USA; mhderbala@mdanderson.org (M.H.D.); bastephen@mdanderson.org (B.S.); fmeric@mdanderson.org (F.M.-B.); 6 Department of Medicine, Section of Hematology/Oncology, West Virginia University, Morgantown, WV 26506, USA; rhnofal@gmail.com

**Keywords:** immunotherapy, pembrolizumab, renal medullary carcinoma, SMARCB1

## Abstract

**Simple Summary:**

Renal medullary carcinoma (RMC) is an aggressive type of kidney cancer. Traditional treatments have limited effectiveness, so new strategies are needed. Studies of RMC tissues found signs of inflammation, suggesting that immune checkpoint therapies could be a potential treatment option. In this study, we tested the effectiveness of pembrolizumab, an immune checkpoint inhibitor in a group of patients with RMC. Unfortunately, the results showed that pembrolizumab did not stop tumor growth. All the patients experienced rapid disease progression. One patient had such rapid progression that they had to stop the treatment less than a week after receiving pembrolizumab. In conclusion, this study demonstrated that pembrolizumab did not show any clinical benefits in patients with RMC.

**Abstract:**

Background. Renal medullary carcinoma (RMC) is one of most aggressive renal cell carcinomas and novel therapeutic strategies are therefore needed. Recent comprehensive molecular and immune profiling of RMC tissues revealed a highly inflamed phenotype, suggesting the potential therapeutic role for immune checkpoint therapies. We present the first prospective evaluation of an immune checkpoint inhibitor in a cohort of patients with RMC. Methods. A cohort of patients with locally advanced or metastatic RMC was treated with pembrolizumab 200 mg intravenously every 21 days in a phase II basket trial (ClinicalTrials.gov: NCT02721732). Responses were assessed by irRECIST. Tumor tissues were evaluated for PD-L1 expression and for tumor-infiltrating lymphocyte (TIL) levels. Somatic mutations were assessed by targeted next-generation sequencing. Results. A total of five patients were treated. All patients had advanced disease, with the majority of patients (60%) having metastatic disease at diagnosis. All patients had rapid disease progression despite pembrolizumab treatment, with a median time to progression of 8.7 weeks. One patient (patient 5) experienced sudden clinical progression immediately after treatment initiation and was thus taken off trial less than one week after receiving pembrolizumab. Conclusions. This prospective evaluation showed no evidence of clinical activity for pembrolizumab in patients with RMC, irrespective of PD-L1 or TIL levels.

## 1. Introduction

First described as a distinct entity in 1995 [1], renal medullary carcinoma (RMC) is a rare type of kidney cancer that afflicts almost exclusively young patients with a sickle cell hemoglobinopathy, usually of African descent [2,3,4]. RMC is characterized by the loss of the SMARCB1 protein (also known as INI-1, hSNF5, or BAF47). SMARCB1 is a subunit of the Switch/Sucrose Non-Fermentable (SWI/SNF) complex, a tumor suppressor that is a mediator of chromatin remodeling and modulates transcriptional activity [4,5,6,7,8]. Although RMC comprises less than 0.5% of all RCC cases [9], it is the third-most-common kidney malignancy among adolescents and young adults [6] and is highly aggressive, with less than 5% of patients surviving longer than 36 months on current therapies [10,11]. Due to the rarity of RMC, no randomized clinical trials have been conducted for this disease and therapeutic choices are informed by case reports, small patient series and consensus expert opinion [11,12]. Platinum-based cytotoxic chemotherapy is the mainstay of RMC therapy, with a 29% objective response rate noted in a multicenter retrospective study, with only 13% of patients surviving longer than 2 years [10]. RMC has shown a lack of response to vascular endothelial growth factor (VEGF)-directed multi-tyrosine kinase inhibitors (TKIs) and mTOR inhibitors, as well as to other targeted therapies that are used to treat common RCCs subtypes [4,13,14]. Because of the aggressive nature of this disease and poor outcomes, there is great need for new therapeutic strategies [8,15,16,17].

Advances in cancer immunology and the introduction of novel immunotherapy therapies, such as programmed death receptor-1 (PD-1) and programmed death ligand (PD-L1) inhibitors, has produced clinical benefit in numerous solid tumor types [18]. Ongoing efforts to assess the efficacy of these potent therapies in various tumors types include a phase II basket trial evaluating the efficacy of pembrolizumab in patients with rare tumors that are unresectable or metastatic (ClinicalTrials.gov: NCT02721732). Tumor types included in this trial include RMC, carcinomas of unknown primary type, adrenal gland pheochromocytomas, germ cell tumors, paraganglioma, penile carcinomas, squamous cell carcinomas of the skin, small cell carcinomas, granulosa cell tumors of the ovary, and adrenocortical carcinomas. The efficacy of pembrolizumab has been reported in several of the rare solid tumor cohorts enrolled in trials [19,20,21,22,23]. Comprehensive molecular and immune profiling of RMC tissues revealed a highly inflamed phenotype associated with cGAS-STING pathway upregulation and heterogeneous PD-L1 expression in the setting of a low tumor mutational burden [24]. A case report noted a durable response in a patient with RMC following therapy with the PD-1 inhibitor, nivolumab [7]. However, another report noted only brief disease stabilization with nivolumab in a patient with RMC, while a third patient demonstrated disease progression as best response after five infusions of nivolumab monotherapy [25]. Prospective evaluation is therefore warranted to elucidate the efficacy of PD-1 inhibition in RMC. Herein, we report the RMC cohort results of an ongoing phase II clinical trial evaluating the efficacy of the PD-1 inhibitor pembrolizumab in patients with rare tumors.

## 2. Materials and Methods

This is a prespecified cohort from an open-label phase II basket trial of pembrolizumab in patients with advanced rare cancers whose tumors had shown progression within the previous six months. The study took place at the University of Texas MD Anderson Cancer Center and was granted approval by both the US Food and Drug Administration and MD Anderson’s institutional review board. The trial is documented on ClinicalTrials.gov under the identifier NCT02721732, as well as under MD Anderson Protocol ID # 2015-0948. The overall results of non-RMC cohorts in this phase II study were previously reported [21].

### 2.1. Study Design and Participants

Eligible participants were adults aged 18 years and above, diagnosed with advanced RMC via histological confirmation. MDACC was responsible for the assessment of all tumor samples, with the RMC diagnosis verified through the detection of SMARCB1 (INI-1) protein loss using IHC. Other inclusion criteria comprised an Eastern Cooperative Oncology Group (ECOG) performance status of 0 or 1, alongside sufficient organ and bone marrow function. Prior to enrollment, every patient gave their informed consent. Participants received pembrolizumab 200 mg intravenously every 21 days and continued the treatment until either disease progression was recorded, one or more adverse events became intolerable, consent was withdrawn, or the investigator decided to cease the treatment. Grounds for removal from the study included either clinical or radiologic disease progression. Responses were evaluated using the Immune-Related Response Evaluation Criteria In Solid Tumors (irRECIST) [26] guidelines, through serial radiologic imaging (CT scans of the chest, abdomen, and pelvis) at baseline, every 9 weeks for the first half-year, and then every 12 weeks at the discretion of the investigator. Adverse events were rated based on the National Cancer Institute Common Terminology Criteria for Adverse Events, V.4.03 [27].

### 2.2. PD-L1, TIL Scoring, and Somatic Tumor Mutations

Biomarker analyses were performed on fresh tissue samples obtained at baseline or on an archival tissue sample. The PD-L1 expression and tumor-infiltrating lymphocyte (TIL) were determined and scored as previously described [21]. The presence or absence of stromal interface was also assessed via IHC. Somatic mutations were assess using DNA extracted from the sample and analyzed using a targeted next-generation sequencing (NGS)-based analysis developed by the Molecular Diagnostic Laboratory (MDL) at the M.D. Anderson Cancer Center [28]. 

### 2.3. Statistical Analysis

The primary endpoint of the study was to determine the proportion of patients who remained alive and without disease progression at 27 weeks (9 cycles). Secondary endpoints included determining the objective response rate (partial or complete response), a clinical benefit rate of equal to or greater than 4 months (which included complete response, partial response, or stable disease), the correlation between the non-progression rate (NPR) at 27 weeks and the baseline PD-L1 status, as well as assessing the safety and tolerability of the treatment. An additional exploratory aim was to assess the potential role of TILs in predicting the effectiveness of the therapy.

The trial used Simon’s optimal two-stage design for each cohort. In the first stage, 12 patients were enrolled. If three or more of these patients achieved the primary endpoint (no disease progression at 27 weeks), then the study would proceed to the second stage, which involved the recruitment of an additional 13 patients.

Descriptive statistical methods were employed to present the patient characteristics. Patients were included in the outcome analysis provided that they had at least one adequate on-study tumor assessment, and were included in the safety analysis if they had received a minimum of one dose of pembrolizumab. The best overall response was defined as the best response recorded from the initiation of the treatment until either the disease progressed or the treatment was discontinued for any reason. Waterfall plots were used to illustrate the best overall response per irRECIST.

## 3. Results

We enrolled and evaluated five patients in the RMC cohort. The baseline clinical characteristics and tumor characteristics are summarized in Table 1 and Table 2. Patients in this study were predominantly male (4 out 5) in keeping with the known male predominance in this disease. Four of the five patients were in their 20s (median age 24) and only one patient was 47 years old. Of note, the 47-year-old patient was the only female in the cohort. Three patients self-identified as African American, one patient identified as non-Hispanic white and the ethnicity background of the last patient is unknown. All patients in this cohort had the sickle cell trait. Hemoglobin electrophoresis data were available for 3 of the 5 patients and confirmed the presence of the sickle cell trait status. Three of the five primary tumors occurred in the right kidney. At enrollment, all patients had an ECOG performance status of 0 or 1. All patients had advanced disease at diagnosis with the majority of the patients (3 of 5) having metastatic disease at initial diagnosis and the rest had a Stage III disease. Patients had received 0 to 2 prior lines of therapy before receiving pembrolizumab (Table 3). Four of the five patients had nephrectomy prior to initiation of any systemic therapy.

**Figure 1:**
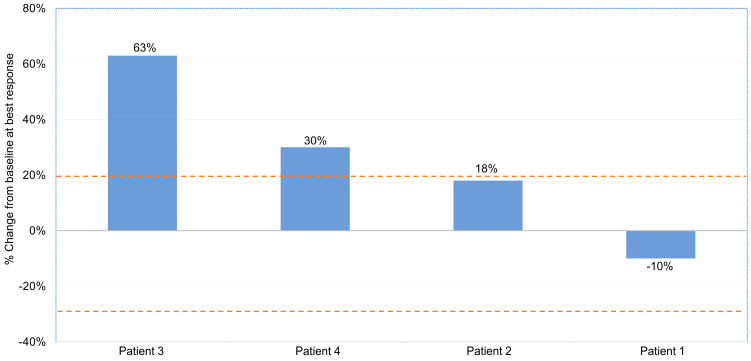
Waterfall plot illustrating the best objective response to pembrolizumab in four patients (Patients 1–4) as per the immune-related Response Evaluation Criteria in Solid Tumors (irRECIST) criteria. The fifth patient experienced clinical progression prior to restaging imaging. The area beneath the lower orange dotted line denotes a partial response (a decrease in the sum of the diameters of the target lesions by 30% or more compared to the baseline). The area between the two orange dotted lines indicates stable disease. The area above the upper orange dotted line signifies progressive disease (an increase in the sum of the diameters of target lesions by 20% or more, compared to the smallest sum during the study), based on irRECIST.

**Figure 2:**
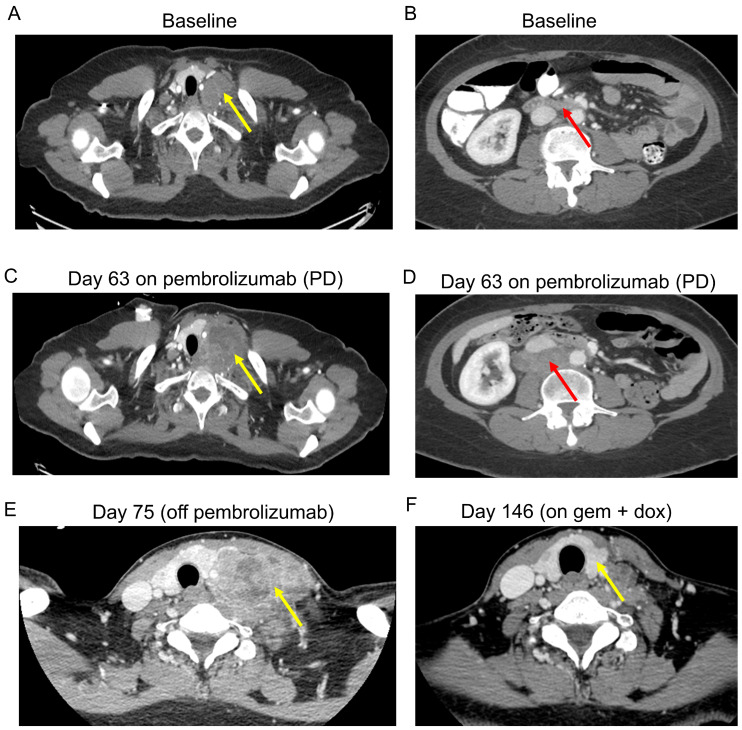
Patient 3 in the trial presented with gross hematuria and was found to have a 4.3 cm left renal mass with retroperitoneal adenopathy for which the patient underwent a left nephrectomy. Twenty days later, presented to MDACC with progressive disease in the retroperitoneum. Initiated on treatment with paclitaxel, carboplatin and bevacizumab and had disease progression after 5 cycles. Subsequently enrolled on a clinical trial with pembrolizumab. Panels (**A**,**B**) show disease burden in the mediastinum (yellow arrows) and retroperitoneal lymph nodes (red arrows) prior to initiation of pembrolizumab. The patient had radiographic (**C**–**E**) and symptomatic disease progression (PD) within 9 weeks from initiation of pembrolizumab and was urgently hospitalized 7 days after discontinuation for management of severe pain due a rapidly enlarging neck mass (**E**), back pain and syncopal episodes induced by the pressure of the mass against the carotid body. Emergently started on gemcitabine (gem) 900 mg/m^2^ over 90 min and doxorubicin (dox) 40 mg/m^2^ with rapid improvement in symptoms and radiographic response (**F**).

Of the 5 patients, 4 patients had complete imaging data for assessment using irRECIST criteria, and the results are displayed in Table 4 and Figure 1. All patients had aggressively rapid disease progression despite pembrolizumab treatment, with a median time to progression of 8.7 weeks after initiation of therapy, with two out of four patients (patient 3 and patient 4) fulfilling established radiological criteria for a hyperprogressive disease [29]. No imaging review was performed in the trial of one patient (patient 5) who had clinical progression immediately after starting treatment and was taken off trial less than one week after receiving pembrolizumab in order to start a different therapy. Notably, this was the only patient who did not have any cytoreductive surgery and had extensive metastatic disease at trial initiation. Time to disease progression (TTP) ranged from 0.7 to 12.7 weeks from first treatment, with a median TTP of 8.7 weeks. Figure 2 illustrates the treatment progression of patient 3 following the initiation of pembrolizumab treatment, as well as the response to subsequent chemotherapy. There were no objective responses (partial or complete) observed amongst all five patients, and only one patient had transient disease control with stable disease as best response for 13 weeks prior to progression. The remainder of the patients had disease progression as best response. The treatment was relatively well-tolerated, with no grade 3 or higher toxicity. Only one patient was noted to have grade 1 fatigue. No other adverse events were identified. As none of the five patients had met the primary endpoint of non-progression at 27 weeks, enrollment to the second stage was not initiated.

Table 4 list the PD L1 H-scores, TIL scores and results of a targeted NGS analysis. PD-L1 staining in our cohort varied widely from 0 to 45%, with a median of 5% and average of 13.4%. PD-L1 H-score in our cohort varied widely from 0–65, with a median H-score of 5 and average H-score of 18.4. The only patient to attain some disease control (stable disease) had a PD-L1 staining and H-score of 0. TIL infiltration was present in all baseline specimens. Three patients had high TIL infiltrations with a score of 3. One patient had a score of 2 and the other had a score of 1. Somatic tumor mutation gene panel results were available for four of the five patients. Patient 2 was found to have a BRAF V600E mutation and Patient 3 was found to have a PTCH1 (c.4034 g > Ap.R1345H) mutation. Two patients with gene panels (patients 1 and 5) did not have detectable mutations. Patient 4 did not have mutation gene panel performed.

## 4. Discussion

Herein, we report the results of the first prospective study of single-agent pembrolizumab in RMC. The striking lack of objective responses and rapid disease progression led to discontinuation of further enrollment of patients with RMC in this trial. All five patients had rapid and aggressive disease progression in less than 13 weeks from initiation of therapy. It is also notable that patients in this study were relatively treatment-naïve, with three of them having received only one or no prior lines of therapy and two patients having received two prior lines of treatment. 

The heterogeneity of PD-L1 expression and high TIL infiltrates is consistent with the previously reported immune profiling of RMC tissues [24]. PD-L1 status has previously been described as a predictive biomarker of response to PD-1 inhibitor monotherapy in numerous malignancies, such as non-small cell lung cancer and head and neck squamous cell carcinoma [30,31,32]. However, the relationship between PD-L1 expression and response to immune checkpoint therapy has been inconclusive in other malignancies such as small cell lung cancer and clear cell RCC [33]. This is consistent with the present study whereby the only patient to attain some disease control (stable disease) had a PD-L1 staining and H-score of 0, suggesting a discrepancy between PD-L1 scoring and response. This is further supported by the results of Patient 3, who had the highest PD-L1 H-score of 65 but experienced rapid progression with PD at 8.7 weeks (after 3 cycles; first restaging) after the initiation of pembrolizumab treatment.

A recently published comprehensive molecular characterization of RMC showed a robust inflammatory phenotype with high numbers of infiltrating T cells and cytotoxic lymphocytes comparable to those seen in clear cell RCC [24]. However, in contrast to clear cell RCC, RMC tumors were noted to also harbor increased numbers of myeloid dendritic cells, neutrophils, FOXP3+ regulatory T cells, and B lineage cells compared with clear cell RCC [24]. Ongoing studies are being performed to elucidate the specific functional states of TILs and other cells in the immune microenvironment and tumor cell compartment of RMC tissues that may explain the aggressive growth of RMC following treatment with PD-1 inhibitor monotherapy. Notably, we previously reported that RMC tissues upregulated expression of immune-checkpoint receptors such as PD-1, CTLA-4, and LAG3, although PD-L1 expression in tumor cells was notably heterogeneous [24]. It remains to be determined whether inhibition of additional immune checkpoints such as CTLA-4 can produce antitumor responses by activating tumor-reactive effector T cells within the inflamed microenvironment of RMC tissues. A recently completed clinical trial (ClinicalTrials.gov identifier: NCT03274258) evaluated the efficacy of nivolumab in combination with the CTLA-4 inhibitor ipilimumab specifically in patients with RMC with extensive longitudinal blood and tissue collection to elucidate the effects of immune checkpoint therapy in RMC. Another trial (ClinicalTrials.gov identifier: NCT03866382) tested the efficacy of triplet therapy with the tyrosine kinase inhibitor, cabozantinib, in combination with nivolumab and ipilimumab in rare genitourinary malignancies and included an RMC cohort. A recent preclinical study in genetically engineered murine models of RMC found that SMARCB1 loss confers resistance to anti-VEGF TKIs [4]. An ongoing clinical trial (ClinicalTrials.gov identifier: NCT05347212) is currently testing the efficacy of nivolumab in combination with high doses of the LAG-3 inhibitor relatlimab, and is also collecting longitudinal blood and tissue samples to understand the impact of this immune checkpoint inhibitor combination in RMC.

## 5. Conclusions

In conclusion, our prospective evaluation of single-agent pembrolizumab showed no evidence of clinical activity irrespective of PD-L1 or TIL levels. These results suggest that single agent anti-PD1 monotherapy has limited benefit in RMC and that different immunomodulatory strategies may be needed to produce antitumor activity. It is important to note that the non-randomized nature of this study and limited numbers limit our ability to draw definitive conclusions from these results. It will be important to see the results of the combinatorial strategies in ongoing studies, including dual check point inhibitions with nivolumab and ipilimumab or nivolumab and relatlimab.

## Figures and Tables

**Table 1:** Baseline clinical and tumor characteristics for each individual patient.

Patient	Age at C1D1	Gender	Race	Kidney Laterality	ECOG	Hemoglobin Electrophoresis Results	Stage at Initial Diagnosis of RMC	Metastatic Sites
1	24	Male	Caucasian	right	0	Hb AS	Stage III (T3aN1M0)	4 (3 lung nodules; T10 lesion)
2	23	Male	Black	Left	1	unavailable	Stage III (T3aNxMx)	Bilateral lung nodules
3	47	Female	Black	left	1	Unavailable	Stage IV (T1bNxM1) thoracic LN met	Local recurrence; Supraclavicular and RP LN
4	23	Male	Black	right	1	Hb AS	Stage IV (T1 N1 M1; left lung nodules)	Bilateral lung nodules; bilateral hilar LND; Left acetabular metastasis
5	24	Male	Black	Right	1	Hb AS	Stage IV (T1 N1 M1; 1.7 cm LLL nodule; bone and adenopathy at dx)	LLL nodule; bone metastasis (T2/T9) and RP adenopathy and hilar adenopathy

**Table 2:** Overall clinical, demographic and treatment response characteristics.

Median Age (Range)	24 (23–47)
Gender	
Male (%)	4 (80%)
Female	1 (20%)
ECOG	
0 (%)	1 (20%)
1 (%)	4 (80%
Prior line	
0	1 (20%)
1	2 (40%)
2	2 (40%)
Best response	
Complete response	0
Partial response	0
Stable disease	1 (20%)
Progressive disease	4 (80%)
Duration from C1D1 to first PD	
Range (weeks)	0.7–12.7
Median (weeks)	8.7
Mean (weeks)	7.4
Adverse Effect	
All grades	1 (20%)
Grade 1 or 2	1 (20%)
Grade 3, 4 or 5	0
Immune related	0
Status at time of trial end (9/30/20)	
Alive	1 (20%)
Deceased	4 (80%)
PD-L1 H score *	
range	0–65
Median	5
Mean	18.5
Modified Proportion score **	
range	0–65
Median	5
Average	13.4
Stromal interface	
Yes	2 (40%)
No	(60%)
	3

* Modified proportion score is obtained by the addition of proportion of PD-LI stained at the difference staining levels (1+, 2+, 3+) *. ** H Score is a semiquantitative score obtained by multiplying the proportion of stain at each stain level and add adding the total: (3 × “% of 3+ cells”) + (2 × “% of 2+ cells”) + (1 × “% of 1+ cells”).

**Table 3:** Prior treatments before pembrolizumab.

Patient	Prior Cytoreductive Therapy	No. of Prior Systemic Therapy	No Platinum Base Therapy	Prior Systemic Therapy
1	Yes	1	0	1. Tazemetostat
2	Yes	2	1	1. Tazemetostat 2. Carboplatin/Gemcitabine/Paclitaxel
3	Yes	2	2	1. Carboplatin + Paclitaxel 2. Carboplatin + Paclitaxel + Avastin
4	Yes	0	0	No prior lines.
5	No	1	0	1. Ixazomib + Adriamycin + gemcitabine

**Table 4:** Response to pembrolizumab.

Patient	Overall Response by irRECIST at Best Response (% Change from Baseline)	Duration from C1D1 to First PD/Last Response Assessment (Weeks)	PD-LI H-Score	MPS	TIL Infiltration (0–3)	NGS (Mutations Identified)
1	10% decrease + non-target PD	8.9	5	5	3	Yes (No actionable mutations)
2	18% increase	12.7	0	0	2	Yes (BRAFV600E)
3	61% increase	8.7	65	45	3	Yes (PTCH1 mutation)
4	30% increase	6.1	2	2	1	No
5	Clinical progression	0.7	20	15	3	Yes (No identified mutations)

## Data Availability

The data presented in this study are available on request from the corresponding authors.
